# Risk of neoplasms with semaglutide in patients with type 2 diabetes: a systematic review and meta-analysis of randomized controlled trials

**DOI:** 10.3389/fphar.2026.1916932

**Published:** 2026-09-03

**Authors:** Yuanyuan Zhong, Xing Tan, Ling Zhang, Cangui Wu

**Affiliations:** 1 Department of Pharmacy, The Third People’s Hospital of Yunnan Province, The Second Affiliated Hospital of Dali University, Kunming, Yunnan, China; 2 Department of Breast Oncology, Jieyang People’s Hospital, Jieyang, Guangdong, China

**Keywords:** GLP-1 receptor agonists, neoplasms risk, randomized controlled trials, semaglutide, type 2 diabetes

## Abstract

**Background:**

Semaglutide is prescribed in conjunction with diet and exercise to improve glycemic control in adults with type 2 diabetes (T2DM). However, its potential impact on tumorigenesis has prompted considerable debate.

**Methods:**

The meta-analysis was conducted in accordance with PRISMA 2020 guidelines. We searched Web of Science, PubMed, Embase, and ScienceDirect for eligible randomized controlled trials (RCTs). The outcome was the risk of overall, benign and malignant neoplasms. The pooled results were expressed as odds ratio (OR) with 95% confidence interval (95% CI). Influential publication was determined by performing the sensitivity analysis. Publication bias was assessed using the funnel plot, Begg’s and Egger’s tests.

**Results:**

Nineteen RCTs conducted between 2016 and 2025 were included, enrolling of 38,160 patients with T2DM. The interventions involved subcutaneous semaglutide and oral semaglutide, with treatment durations ranging from 26 to 156 weeks. Fixed-effects meta-analysis revealed that semaglutide use was associated with an increased risk of overall neoplasms (OR = 1.19, 95% CI = 1.07 to 1.32, *P* = 0.001) and benign neoplasms (OR = 1.28, 95% CI = 1.02 to 1.61, *P* = 0.032), but showed no significant association with malignant neoplasms (OR = 1.12, 95% CI = 1.00 to 1.26, *P* = 0.054). No influential publications or significant publication bias were detected.

**Conclusion:**

Semaglutide use in patients with T2DM is associated with a modestly increased risk of overall and benign neoplasms, but not malignant neoplasms. These findings highlight the importance of maintaining clinical awareness regarding potential neoplastic risks, and further long-term RCTs are warranted to clarify their clinical significance.

**Systematic Review Registration:**

https://www.crd.york.ac.uk/PROSPERO/view/CRD420261354386, Identifier CRD420261354386.

## 1 Introduction

Type 2 diabetes mellitus (T2DM) constitutes a pervasive systemic metabolic disorder and represents a critical global public health challenge. According to projections by the International Diabetes Federation, the global population affected by T2DM is anticipated surging to 783 million by 2045 (Sun et al., 2022). Obesity and diabetes are inextricably linked physiologically. This comorbidity significantly amplifies the risk of severe cardiovascular sequelae, including atherosclerosis and hypertension. Consequently, this situation drives elevated mortality rates and imposes a profound socioeconomic and healthcare burden globally (Joseph et al., 2022).

Given the inherent progression of the pathology, many individuals managed with basal insulin eventually require an escalation of their treatment regimen. This strategic intensification is pivotal for maintaining rigorous glucose control and mitigating the incidence of associated complications (Bailey and Kodack, 2011). Glucagon-like peptide-1 (GLP-1) is an incretin hormone secreted by intestinal L-cells that mediates glucose-dependent insulin release. Concurrently, it exerts several ancillary metabolic effects, including the suppression of glucagon release, delayed gastric emptying, and the promotion of satiety (Santella et al., 2020). GLP-1receptor agonists (GLP-1RAs) have emerged as a cornerstone in the management of T2DM, distinguished by their dual capacity to lower glucose levels while facilitating weight reduction and ensuring mitigated risk of hypoglycemia (Lee et al., 2024). T2DM is widely recognized as the principal cause of chronic kidney disease in a multitude of countries. The use of insulin and sulfonylureas is associated with concomitant weight gain and an elevated susceptibility to hypoglycemia across the general patient population, with a significantly heightened risk observed in patients with chronic kidney disease (Abdi et al., 2018). Conversely, the GLP-1Ras, such as semaglutide, liraglutide, albiglutide, and dulaglutide, do not necessitate dose modification in patients with chronic kidney disease, thereby representing viable therapeutic alternatives to other glucose-lowering agents (Davies et al., 2016). A consensus across recent endocrine and cardiovascular treatment protocols has endorsed GLP-1RAs as a preferred second-line modality for the management of adult patients with T2DM (Davies et al., 2018). In addition to optimizing glucose homeostasis, certain agents in these classes show a pleiotropic capacity to attenuate the risk of major cardiovascular events and chronic kidney disease progression, while concurrently mitigating hypoglycemia susceptibility and facilitating the weight reduction (Yin et al., 2020).

Semaglutide, a long-acting GLP-1 analogue, is prescribed alongside diet and exercise to enhance glycemic management in adults with T2DM, offering either weekly subcutaneous or daily oral administration (Arod et al., 2023). Robust evidence derived from the SUSTAIN and PIONEER clinical trial programmes underpinned the approval of semaglutide in European markets for T2DM. These comprehensive studies evaluated semaglutide against placebo and active comparators across a broad patient demographic, effectively encompassing the full continuum of care (Tilinca et al., 2021). During the SUSTAIN programme, semaglutide has undergone comprehensive evaluation through head-to-head comparisons against a spectrum of antidiabetic agents, assessed both as a standalone therapy and in conjunction with other glucose-lowering medications (Gaede et al., 2019). Among once-weekly GLP-1RAs, subcutaneous semaglutide demonstrates superior efficacy regarding glycemic control and weight reductions relative to sitagliptin and dulaglutide (Ji et al., 2021; Pratley et al., 2018).

Given the widespread clinical application of GLP-1RAs in managing T2DM, their potential impact on tumorigenesis prompted considerable debate. Several meta-analyses have indicated that GLP-1RA use is not associated with an increased risk of malignancy (Cao et al., 2019; Liu et al., 2019), but these findings are juxtaposed with reports indicating an increased risk of benign and malignant neoplasms was associated with liraglutide in comparison to other GLP-1Ras (Yang et al., 2022). Additionally, GLP-1RAs are contraindicated in patients with a personal or family history of specific thyroid malignancies. This restriction is based on preclinical evidence demonstrating that GLP-1RAs induce dose- and duration-dependent tumors in animals, despite the uncertain translational relevance to humans (Elashoff et al., 2011). Moreover, retrospective analysis of the Food and Drug Administration (FDA) database indicated exenatide use was associated with a greater incidence of reported thyroid and pancreatic cancer cases than rosiglitazone use (Elashoff et al., 2011). Therefore, a comprehensive investigation into the risk of neoplasms associated with semaglutide exposure is warranted to ensure patient safety and guide clinical decision-making.

## 2 Methods

The study was in line with Preferred Reporting Items for Systematic Reviews and Meta-Analysis (PRISMA) guidelines (Page et al., 2021; Stroup et al., 2000). The protocol for this systematic review and meta-analysis was registered on PROSPERO (ID: CRD420261354386).

### 2.1 Eligibility criteria

RCTs on comparing the risk of overall, benign and malignant neoplasms with semaglutide in patients with T2DM will be included. Overall neoplasms were defined as the sum of all neoplastic events reported. If a study provided a total count of neoplasms without subclassification, it was included in the overall neoplasms. Benign and malignant neoplasms were extracted directly as reported. Studies that meet one of the following criteria were excluded: (1) laboratory-based research; (2) non-English language; or (3) full text unavailable.

### 2.2 Search strategy

The literature search was conducted in accordance with the PICO framework, focusing on patients with T2DM (P), semaglutide (I), and the risk of neoplasms (O). The Web of Science, PubMed, ScienceDirect and Embase databases were screened up to April, 2026. Besides, a manual search of reference lists from relevant reviews and meta-analyses was performed to identify eligible studies. The search strategy utilized the following query: [Semaglutide and (Type 2 diabetes or T2DM or Diabetes mellitus) and (Neoplasm or Tumor* or Adenoma or Cancer*)].

### 2.3 Study selection

After the retrieval of studies from the databases, records were deduplicated using EndNote software. Two reviewers independently screened the titles and abstracts for relevance to the study objectives. Finally, the full texts of potentially eligible studies were assessed against the inclusion and exclusion criteria. Any discrepancies among the reviewers were resolved through discussion or by consulting a third reviewer.

### 2.4 Quality assessment

Two reviewers independently assessed the risk of bias for the included RCTs using the version 2 of the Cochrane risk-of-bias (RoB 2) tools (Nejadghaderi et al., 2024; Sterne et al., 2019). Any discrepancies were resolved by consulting a third reviewer.

### 2.5 Data extraction and synthesis

Data extraction was performed independently by two reviewers, with discrepancies resolved through discussion or adjudication by a third reviewer. A standardized data collection form was used to extract the following variables: first author, publication year, country, trail code, treatment duration, sample size, mean age, and usage of semaglutide. The pooled results were expressed as odds ratio (OR) with 95% confidence interval (95% CI). Statistical heterogeneity was evaluated using the I^2^ statistic and Cochran’s Q test, where the values of 25%, 50%, and 75% were interpreted as low, moderate, and high heterogeneity, respectively. A random-effects model was employed when moderate to high heterogeneity was detected; otherwise, a fixed-effects model was applied. Influential publication was determined by performing sensitivity analysis. In addition, potential source of heterogeneity was identified by subgroup analysis. Publication bias was assessed by using the funnel plot, Begg’s and Egger’s tests.

### 2.6 Statistical analysis

Statistical analyses were performed using Stata 16 software. The “metan,” “metaninf,” “metafunnel,” and “metabias” commands were utilized to conduct the pooled analyses, sensitivity analyses, and publication bias assessments, respectively. All statistical tests were two-tailed, with a *P*-value < 0.05 considered statistically significant.

## 3 Results

This study presents a comprehensive meta-analysis evaluating the risk of overall, benign and malignant neoplasms associated with semaglutide use in patients with T2DM, thereby providing valuable reference for clinical medication decisions.

### 3.1 Study selection

**FIGURE 1:**
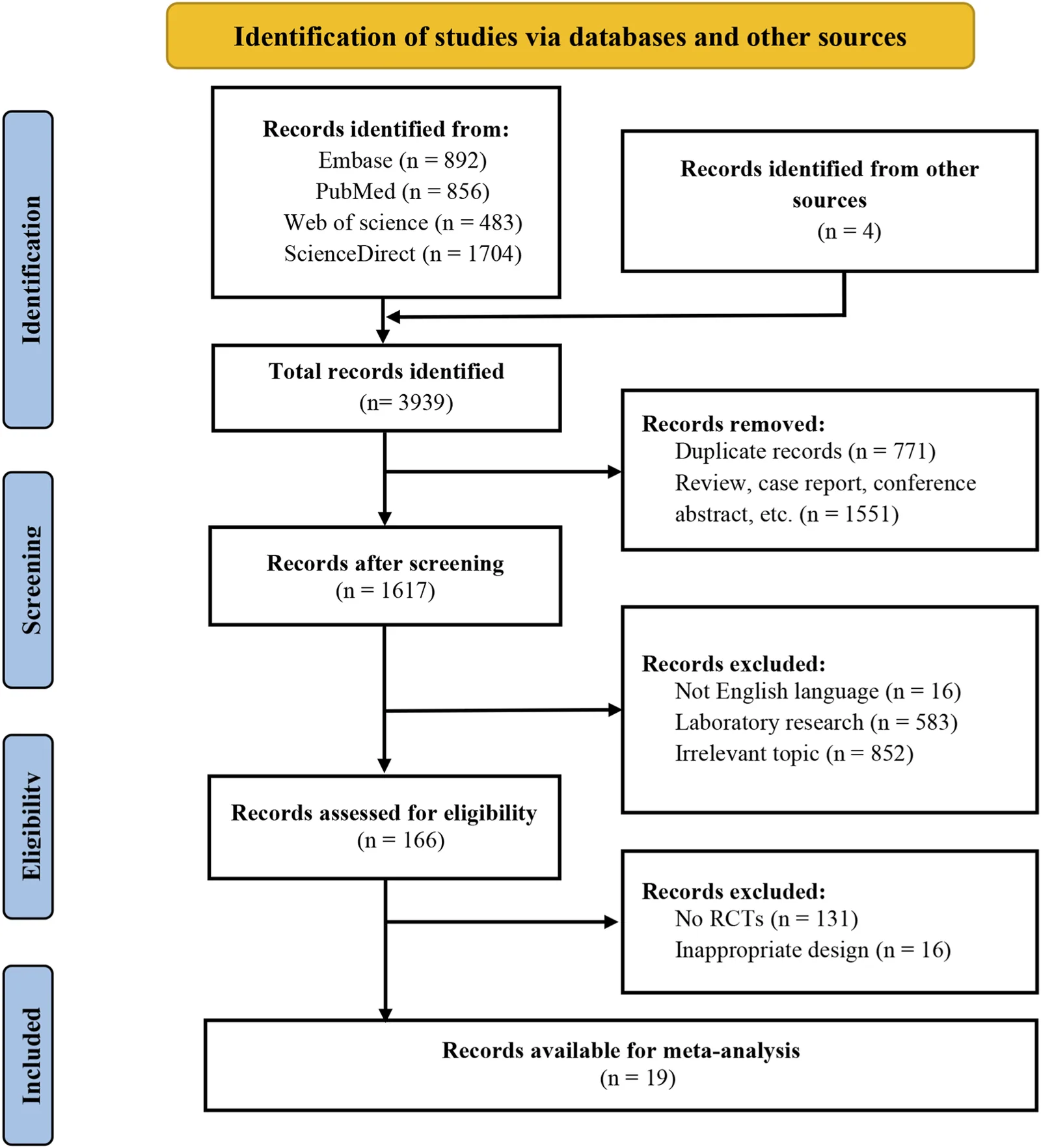
PRISMA flowchart of 19 included RCTs.

The PRISMA flow diagram for study selection is presented in Figure 1. The literature search yielded 3,935 records, with four additional studies identified manually. After removing 771 duplicates and screening out 1,551 irrelevant records, 1,617 records underwent title/abstract screening. Subsequently, 1,448 studies were excluded based on language, study design, or topic, resulting in the assessment of 166 full-text articles. Finally, 19 RCTs were eligible for inclusion in the analysis (Aroda et al., 2023; Davies et al., 2021; Davies et al., 2025; Husain et al., 2019; Kadowaki et al., 2025; Lingvay et al., 2025; Mahaffey et al., 2025; Marso et al., 2016; McGuire et al., 2025; Mosenzon et al., 2019; Mu et al., 2024; Perkovic et al., 2024; Pratley et al., 2019; Rodbard et al., 2018; Sorli et al., 2017; Warren et al., 2018; Yamada et al., 2020; Zinman et al., 2019a; Zinman et al., 2019b).

### 3.2 Study characteristics and quality assessment

The 19 included RCTs were published between 2016 and 2025, enrolling a total of 38,160 patients with T2DM. Sample sizes varied from 195 to 9,650 participants, with a mean age ranging from 49 to 70 years. The interventions consisted of subcutaneous semaglutide (12 studies) and oral semaglutide (8 studies) compared to placebo, with treatment durations spanning from 26 to 156 weeks. A comprehensive summary of the study characteristics is provided in Table 1.

**TABLE 1:** Summarized characteristics of 19 included RCTs.

Author, year	Country	Trail code	Duration (week)	Mean age (year)	Usage of semaglutide	Simple size	Outcome
Aroda et al. (2023)	United States	SUSTAIN and PIONEER (NCT03819153)	30	57	Subcutaneous semaglutide: 0.5 mg or 1.0 mg once weekly; Oral semaglutide: 3 mg, 7 mg or 14 mg once-daily	4,807	③
Davies et al. (2021)	Denmark	STEP 2 (NCT03552757)	68	55	Subcutaneous semaglutide: 1.0 mg or 2.4 mg once weekly	1,207	③
Davies et al. (2025)	Denmark	STEP 2 (NCT05394519)	68	56	Subcutaneous semaglutide: 2.4 mg once weekly	1,206	①, ②, ③
Husain et al. (2019)	Canada	PIONEER 6 (NCT03811561)	69	66	Oral semaglutide: 14 mg once daily	3,183	③
Kadowaki et al. (2025)	Japan	OASIS 2 (NCT05132088)	68	49	Oral semaglutide: 50 mg once daily	200	①
Lingvay et al. (2025)	United States	STEP UP T2D (NCT05649137)	72	56	Subcutaneous semaglutide: 7.2 mg or 2·4 mg once weekly	512	①, ②, ③
Mahaffey et al. (2025)	United States	FLOW (NCT03819153)	156	67	Subcutaneous semaglutide: 1 mg once weekly	3,532	③
Marso et al. (2016)	United Kingdom	SUSTAIN 6 (NCT01720446)	104	65	Subcutaneous semaglutide: 0.5 mg or 1.0 mg once weekly	3,297	①, ②, ③
McGuire et al. (2025)	United States	PIONEER 6 NCT03914326()	104	66	Oral semaglutide: 14 mg once daily	9,650	①, ③
Mosenzon et al. (2019)	United States	PIONEER 5 (NCT02827708)	26	70	Oral semaglutide: 14 mg once daily	324	③
Mu et al. (2024)	United Kingdom	SUSTAIN 1 (NCT02054897)	30	54	Subcutaneous semaglutide: 0.5 mg or 1.0 mg once weekly	387	①, ②, ③
Perkovic et al. (2024)	Australia	FLOW (NCT03819153)	104	67	Subcutaneous semaglutide: 1.0 mg once weekly	3,533	③
Pratley et al. (2019)	Germany	PIONEER 4 (NCT02863419)	52	56	Oral semaglutide: 14 mg once daily	427	①, ③
Rodbard et al. (2018)	United States	SUSTAIN 5 (NCT02305381)	30	59	Subcutaneous semaglutide: 0.5 or 1.0 mg once weekly	396	①, ②, ③
Sorli et al. (2017)	China	STEP 7 (NCT04251156)	44	41	Subcutaneous semaglutide: 2.4 mg once weekly	375	①
Warren et al. (2018)	Denmark	SUSTAIN 1–5 (NCT02054897)	40	56	Subcutaneous semaglutide: 0.5 mg or 1.0 mg once weekly	3,898	①, ②, ③
Yamada et al. (2020)	Japan	PIONEER 9 (NCT03018028)	26	60	Oral semaglutide: 3 mg, 7 mg, or 14 mg once daily	195	③
Zinman et al. (2019b)	Canada	SUSTAIN 9 (NCT03086330)	30	57	Subcutaneous semaglutide: 1.0 mg once weekly	301	①
Zinman et al. (2019a)	Canada	PIONEER 8 (NCT03021187)	52	61	Oral semaglutide: 3 mg, 7 mg, or 14 mg once daily	730	③

①, Overall neoplasms; ②, Benign neoplasms; ③, Malignant neoplasms.

The methodological quality was generally high, with 16 studies assessed as taking a low risk of bias. However, three studies were rated as having “some concerns” regarding their overall risk of bias. Specifically, the studies by Mahaffey et al. (2025) and Yamada et al. (2020) raised concerns in the domain of missing outcome data. The study by Perkovic et al. (2024) was flagged for “some concerns” in the domain of selective reporting of results. No studies were found to be at high risk of bias and was excluded (Supplementary Figure S1).

### 3.3 The risk of neoplasms associated with semaglutide in patients with T2DM

#### 3.3.1 Semaglutide use was associated with an increased risk of overall neoplasms

**FIGURE 2:**
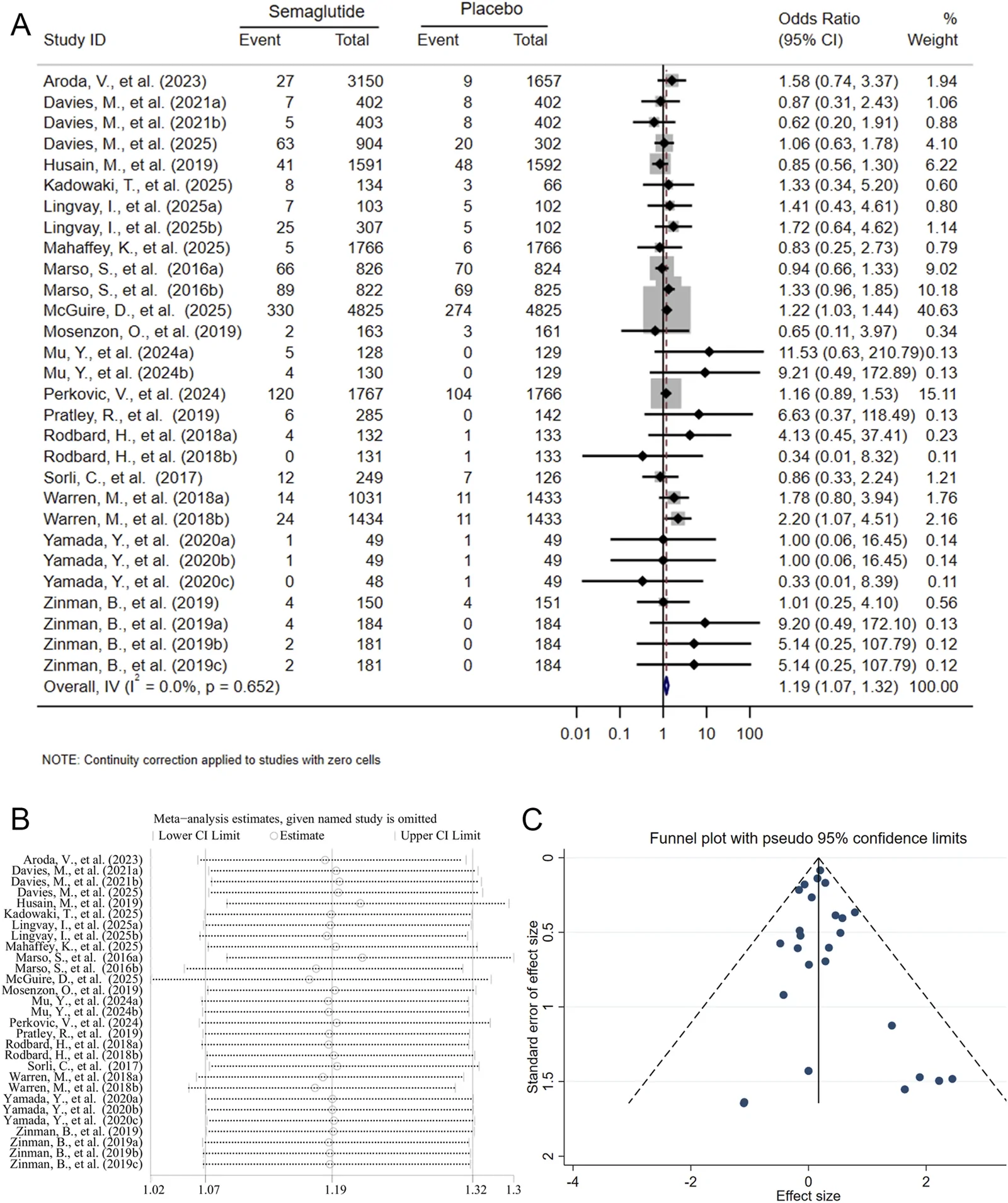
Semaglutide use was associated with an increased risk of overall neoplasms in patients with T2DM. **(A)** Forest plot of the risk of overall neoplasms associated with semaglutide use; **(B)** Sensitivity analysis for the risk of overall neoplasms; **(C)** Funnel plot for assessing publication bias.

A fixed-effects meta-analysis of 19 studies (Aroda et al., 2023; Davies et al., 2021; Davies et al., 2025; Husain et al., 2019; Kadowaki et al., 2025; Lingvay et al., 2025; Mahaffey et al., 2025; Marso et al., 2016; McGuire et al., 2025; Mosenzon et al., 2019; Mu et al., 2024; Perkovic et al., 2024; Pratley et al., 2019; Rodbard et al., 2018; Sorli et al., 2017; Warren et al., 2018; Yamada et al., 2020; Zinman et al., 2019a; Zinman et al., 2019b; Brauner et al., 2006; De Santis et al., 2004; Hashimoto et al., 2021; Ho et al., 2009; Kaufman et al., 2024; Mendez- et al., 2012; Razeghinejad and Nowroozzadeh, 2010) revealed that the administration of semaglutide in patients with T2DM was associated with a statistically significant increase in the risk of overall neoplasms (OR = 1.19, 95% CI = 1.07 to 1.32, *P* = 0.001). The analysis exhibited negligible heterogeneity (I^2^ = 0.0%, *P* = 0.673) (Figure 2A). Sensitivity analysis confirmed that the pooled estimate was robust and not disproportionately driven by any single study (Figure 2B). Moreover, assessments for publication bias yielded no statistically significant results (Figure 2C; Begg’s test: z = 1.03, *P* = 0.302; Egger’s test: t = 1.28, *P* = 0.202).

#### 3.3.2 Semaglutide use was associated with a higher incidence of benign neoplasms

**FIGURE 3:**
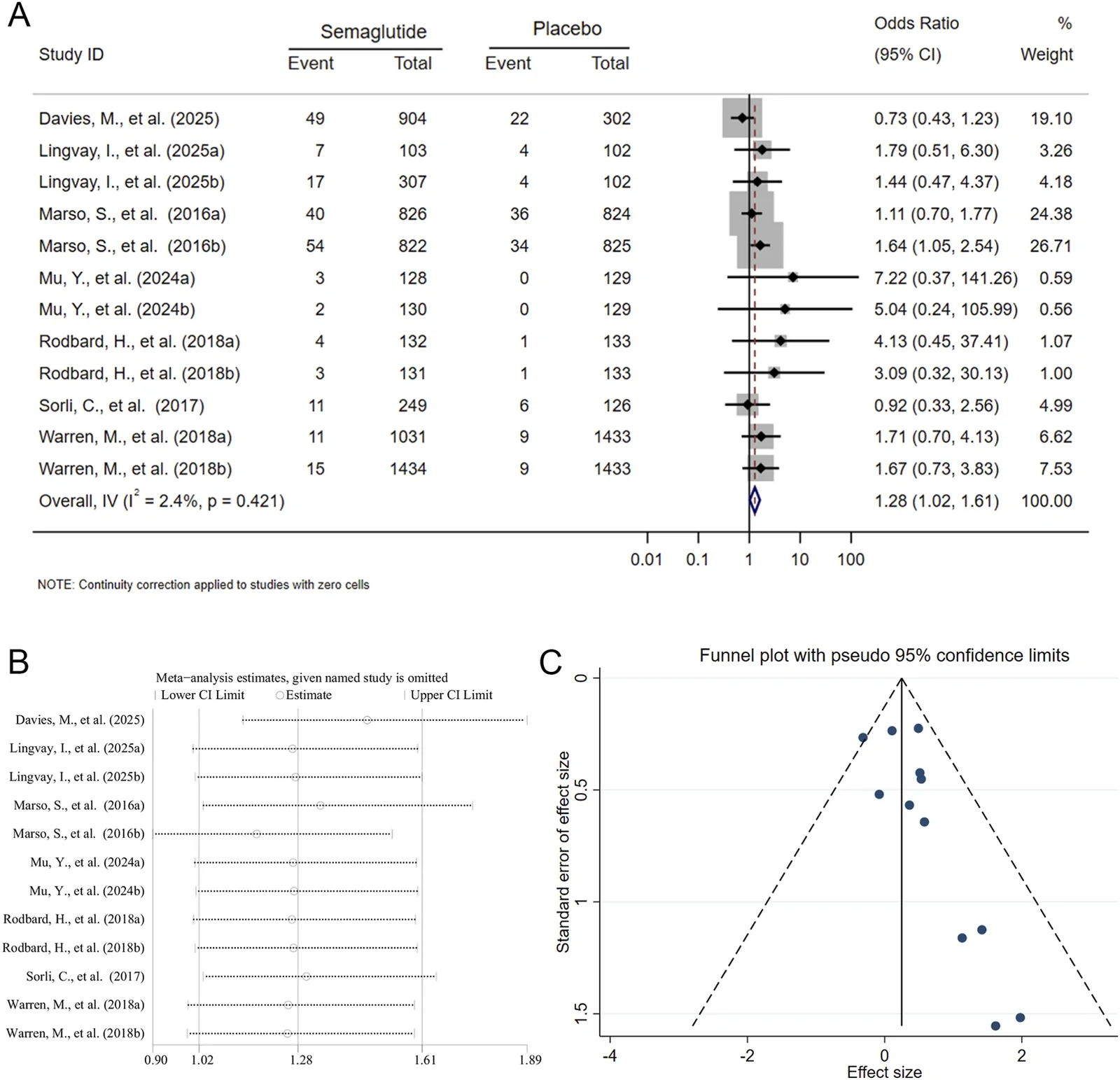
Semaglutide use was associated with a higher incidence of benign neoplasms in patients with T2DM. **(A)** Forest plot of the risk of benign neoplasms associated with semaglutide use; **(B)** Sensitivity analysis for the risk of benign neoplasms; **(C)** Funnel plot for assessing publication bias.

A fixed-effects meta-analysis of seven studies (Davies et al., 2025; Lingvay et al., 2025; Marso et al., 2016; Mu et al., 2024; Rodbard et al., 2018; Sorli et al., 2017; Warren et al., 2018) demonstrated a statistically significant increase in the risk of benign neoplasms among semaglutide users (OR = 1.28, 95% CI = 1.02 to 1.61, *P* = 0.032). This analysis exhibited low statistical heterogeneity (I^2^ = 2.4%, *P* = 0.421) (Figure 3A). Sensitivity analysis confirmed the robustness of the pooled estimate, as no individual study exerted undue influence on the overall effect (Figure 3B). Furthermore, no significant publication bias (Figure 3C; Begg’s test: z = 0.21, *P* = 0.837; Egger’s test: t = 1.19, *P* = 0.235) were detected.

#### 3.3.3 No significant association between semaglutide exposure and the incidence of malignant neoplasms

**FIGURE 4:**
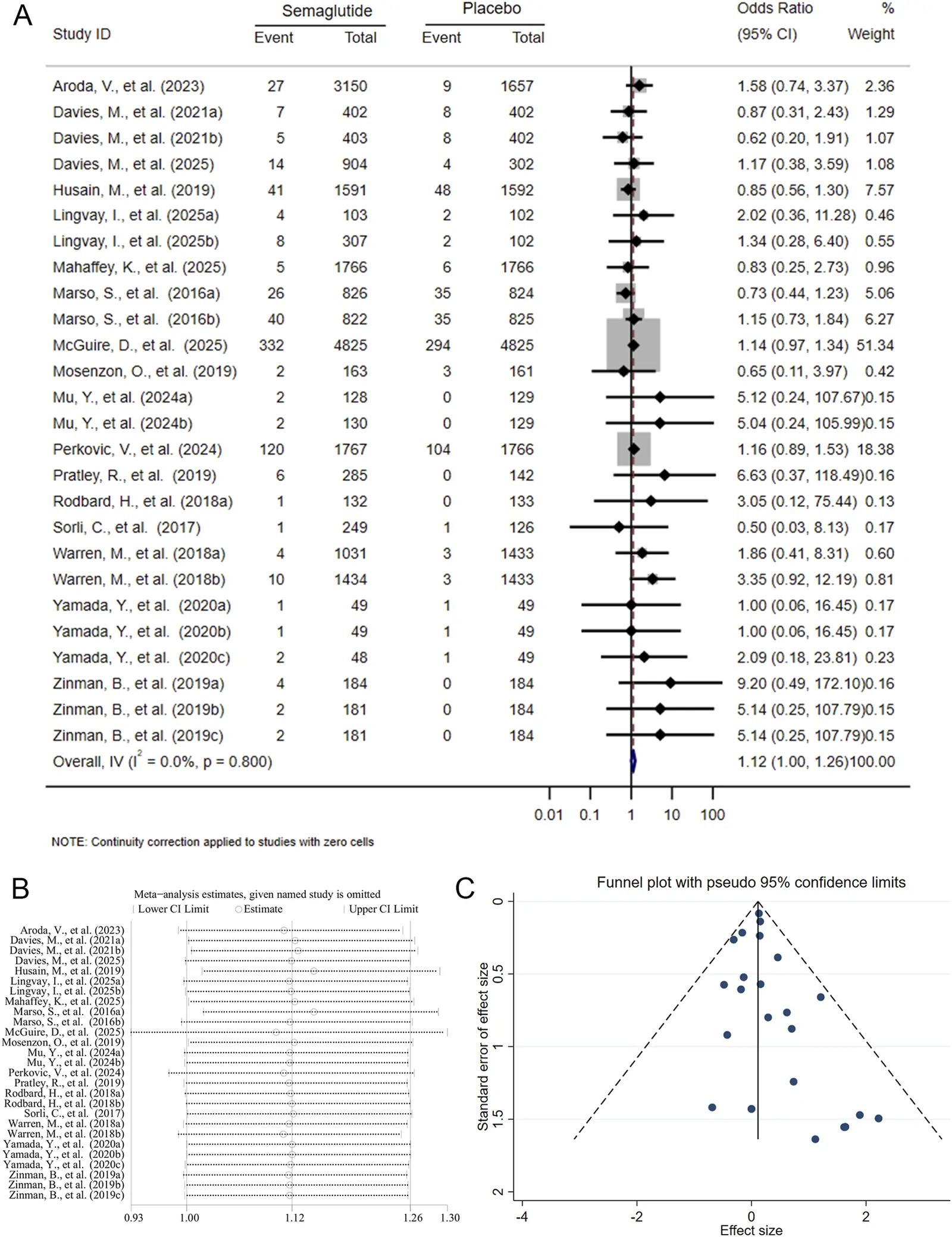
No significant association between semaglutide exposure and the incidence of malignant neoplasms. **(A)** Forest plot of the risk of malignant neoplasms associated with semaglutide exposure; **(B)** Sensitivity analysis for the risk of malignant neoplasms; **(C)** Funnel plot for assessing publication bias.

A fixed-effects meta-analysis of 16 studies (Aroda et al., 2023; Davies et al., 2021; Husain et al., 2019; Lingvay et al., 2025; Mahaffey et al., 2025; Marso et al., 2016; McGuire et al., 2025; Mosenzon et al., 2019; Mu et al., 2024; Perkovic et al., 2024; Pratley et al., 2019; Rodbard et al., 2018; Sorli et al., 2017; Warren et al., 2018; Yamada et al., 2020; Zinman et al., 2019a; Brauner et al., 2006; De Santis et al., 2004; Hashimoto et al., 2021; Kaufman et al., 2024) revealed a non-significant elevation in the risk of malignant neoplasms associated with semaglutide exposure (OR = 1.12, 95% CI = 1.00 to 1.26, *P* = 0.054). The analysis was characterized by very low heterogeneity (I^2^ = 0.0%, *P* = 0.800) (Figure 4A). Influence analysis indicated that no single study disproportionately affected the pooled estimate (Figure 4B). Furthermore, evaluation for small-study effects showed no evidence of significant publication bias (Figure 4C; Begg’s test: z = 1.50, *P* = 0.134; Egger’s test: t = 1.31, *P* = 0.190).

## 4 Discussion

GLP-1RAs, including exenatide, liraglutide, dulaglutide, and semaglutide, are widely employed as second-line or later-line therapeutic agents in the management of T2DM (Drucker, 2002). These agents exert multiple beneficial effects, for patients with diabetes, characterized by the augmentation of glucose-dependent insulin secretion, the attenuation of gastric emptying, and the suppression of appetite and subsequent caloric consumption (Liu, 2024). In particular, the introduction of semaglutide conferred substantial clinical benefits: eliciting greater improvements in glycemic indices and weight management compared to placebo, which underscores its favorable metabolic profile in this population (Rodbard et al., 2018). However, the clinical utility of these agents must be weighed against their safety profile. Gastrointestinal disturbances were the predominant adverse events of semaglutide (Garber, 2011). In terms of formulation-specific safety, the incidence of adverse events was marginally higher with oral semaglutide compared to subcutaneous liraglutide, its safety and tolerability profile remained concordant with the established safety profile of the GLP-1 receptor agonist class (Pratley et al., 2019).

It is well-established that patients with T2DM have a higher baseline risk of developing malignancies. A large-scale study demonstrated that patients with T2DM take a significantly higher risk of developing overall cancer (HR = 1.10) and experience increased post-cancer mortality (HR = 1.23) compared to the general population, with particularly elevated hazards for liver, pancreatic, and uterine cancers (Bjornsdottir et al., 2020). Despite the established efficacy of GLP-1RAs, the risk of tumorigenesis remains a critical area of investigation. To date, data derived from the SUSTAIN clinical program indicate that the overall incidence of malignant neoplasms remained consistently low. Notably, no discernible disparities were observed across varying semaglutide dosage regimens (Davies et al., 2021; Lingvay et al., 2025; Marso et al., 2016; Sorli et al., 2017; Yamada et al., 2020). A large-scale systematic review and meta-analysis (pooled data from 90 RCTs involving 124,791 individuals) revealed no significant association between GLP-1RA therapy and the overall incidence of gastrointestinal cancers (Figlioli et al., 2024). Furthermore, analyses of the FDA Adverse Event Reporting System database indicate that GLP-1RAs are generally safe with respect to malignancy risk, showing no signal of an increased risk for overall malignancy attributable to the drug class (Yang et al., 2022).

However, the safety landscape is complex and requires careful contextualization. Although epidemiological data do not support an association between GLP-1RAs and general neoplastic risk. US regulatory labeling mandates a warning for medullary thyroid cancer based on rodent models, despite the absence of such effects in monkeys or humans (Campos and Unger, 2021). Consistent with this, our investigation yielded no evidence of a causal relationship between semaglutide treatment and the manifestation of malignant neoplasms. Rodent studies have demonstrated that the activation of GLP-1 receptors on thyroid parafollicular C cells stimulates cAMP-dependent calcitonin synthesis and secretion. Furthermore, chronic exposure to these agents induces C-cell proliferation, ultimately leading to the development of C-cell adenomas and carcinomas (Bjerre Knudsen et al., 2010). Similarly, treatment with Exendin-4 resulted in significantly intensified pancreatic acinar inflammation, more numerous pyknotic nuclei, and raised serum lipase levels in rats (Rubino et al., 2021). The pathophysiology of pancreatic carcinogenesis associated with GLP-1 RA use is hypothesized to stem from chronic low-grade inflammation and proliferative alterations, which may precipitate mutations in the KRAS and p53 genes (Suryadevara et al., 2022). Conversely, preclinical evidence indicates that GLP-1 RAs can inhibit proliferation, enhance apoptosis, and attenuate inflammation in prostate cancer cells (Skriver et al., 2023). Several meta-analyses have indicated that GLP-1RA use is not associated with an increased risk of malignancy (Cao et al., 2019; Liu et al., 2019). A retrospective study demonstrated that GLP-1 RA use was not associated with an elevated risk of pancreatic cancer over a 7-year follow-up period (Ailawadi et al., 2026).

To the best of our knowledge, this meta-analysis represents the largest study to date investigating the clinically significant association between semaglutide therapy and tumorigenesis risk outcomes derived from RCTs. Our meta-analysis indicated a significant elevation in the risk of overall neoplasms (OR = 1.19, 95% CI = 1.07 to 1.32, *P* = 0.001), which appears to be primarily driven by benign neoplasms (OR = 1.28, 95% CI = 1.02 to 1.61, *P* = 0.032). Notably, we observed no statistically significant association between semaglutide exposure and the incidence of malignant neoplasms (OR = 1.12, 95% CI = 1.00 to 1.26, *P* = 0.054). Regarding the conflicting signals in specific cancer types, the SUSTAIN-6 trial identified an imbalance in breast cancer events between the semaglutide and placebo groups (HR: 1.73, 95% CI: 0.41–7.34) (Marso et al., 2016). Similarly, Rubino et al. (2021) found the incidence of malignancy among obese adults was 1.1% in the semaglutide group, compared with 0.4% in the control group. Although our meta-analysis demonstrates a statistically significant association between semaglutide and an increased risk of overall neoplasms (OR = 1.19), the relative risk increase does not necessarily equate to a substantial absolute risk for individual patients. Given the well-established cardiovascular and metabolic benefits of semaglutide in managing T2DM, this modestly elevated neoplastic risk must be carefully weighed against its proven therapeutic advantages. Thus, the current evidence does not justify immediate treatment discontinuation, but rather highlights the need for sustained clinical vigilance and long-term monitoring. According to the GRADE framework, the overall certainty of evidence regarding the association between semaglutide and neoplasm risk was rated as moderate quality. While the association with an increased risk of benign neoplasms appears robust, the evidence for malignant neoplasms remains inconclusive due to the necessity for longer-term follow-up data.

The robustness of this study lies in its rigorous randomized, double-blind, placebo-controlled design. This methodological rigor is paramount, as it minimizes bias and ensures the integrity of the evaluated outcome. However, several limitations must be acknowledged. A primary constraint was the trial’s limited duration. The follow-up period was insufficient to fully assess long-term safety. Notably, because the incidence of neoplasms had not reached a plateau by the end of the treatment period, longer-term studies are required to determine the sustained effects and safety profile of the intervention. Furthermore, since neoplasms are rarely primary endpoints in these trials, tumor-related data are predominantly derived from adverse event reports lacking specific histological details. Most trials thus categorized neoplasms broadly (benign, malignant, or unspecified) without specifying tumor types or anatomical sites. Therefore, we were unable to perform subgroup analyses to identify which specific neoplasms primarily drove the observed risk.

## 5 Conclusion

Semaglutide is prescribed alongside diet and exercise to improve glycemic control in adults with T2DM. In this population, semaglutide use is associated with a modestly increased risk of overall and benign neoplasms, but not malignant neoplasms. The overall certainty of evidence regarding the association between semaglutide and neoplasm risk was rated as moderate quality. These findings highlight the importance of maintaining clinical awareness regarding potential neoplastic risks, and further long-term RCTs reporting specific neoplasm subtypes are warranted to clarify their clinical significance.

## Data Availability

The original contributions presented in the study are included in the article/Supplementary Material, further inquiries can be directed to the corresponding authors.
